# Retraction Note: Idelalisib promotes Bim-dependent apoptosis through AKT/FoxO3a in hepatocellular carcinoma

**DOI:** 10.1038/s41419-024-07162-y

**Published:** 2024-10-24

**Authors:** Dan Yue, Xun Sun

**Affiliations:** 1 Department of Laboratory Medicine, Shengjing Hospital of China Medical University, Shenyang, China; 2 Department of Immunology, China Medical University, Shenyang, China

Retraction Note to: *Cell Death and Disease* 10.1038/s41419-018-0960-8, published online 17 September 2018

The Editors-in-Chief have retracted this article because it contains data that overlaps with data from the following article, [1], published by Oncogenesis. In addition, an investigation conducted after the publication of the two articles, which were under consideration at the same time by Cell Death & Disease and Oncogenesis, found additional signs which raise concerns about the authorship of the two manuscripts and the circumstances of the research presented in them. The Editors-in-Chief therefore no longer have confidence in the results and conclusions presented in this article.

The authors have not replied to correspondence from the Publisher.
